# Development and Characterization of Novel Floating-Mucoadhesive Tablets Bearing Venlafaxine Hydrochloride

**DOI:** 10.1155/2016/4282986

**Published:** 2016-05-05

**Authors:** Raghvendra Misra, Peeyush Bhardwaj

**Affiliations:** ^1^Department of Pharmacy, Vivek College of Technical Education, Uttar Pradesh 246701, India; ^2^Institute of Pharmacy, Bundelkhand University, Jhansi, Uttar Pradesh 284128, India

## Abstract

The present investigation is concerned about the development of floating bioadhesive drug delivery system of venlafaxine hydrochloride which after oral administration exhibits a unique combination of floating and bioadhesion to prolong gastric residence time and increase drug bioavailability within the stomach. The floating bioadhesive tablets were prepared by the wet granulation method using different ratios of hydroxypropyl methyl cellulose (HPMC K4MCR) and Carbopol 934PNF as polymers. Sodium bicarbonate (NaHCO_3_) and citric acid were used as gas (CO_2_) generating agents. Tablets were characterized for floating properties,* in vitro* drug release, detachment force, and swelling index. The concentration of hydroxypropyl methyl cellulose and Carbopol 934PNF significantly affects the* in vitro* drug release, floating properties, detachment force, and swelling properties of the tablets. The optimized formulation showed the floating lag time 72 ± 2.49 seconds and duration of floating 24.50 ± 0.74 hr. The* in vitro* release studies and floating behavior were studied in simulated gastric fluid (SGF) at pH 1.2. Different drug release kinetics models were also applied. The* in vitro* drug release from tablets was sufficiently sustained (more than 18 hr) and the Fickian transports of the drug from the tablets were confirmed. The radiological evidence suggests that the tablets remained buoyant and altered position in the stomach of albino rabbit and mean gastric residence time was prolonged (more than > 6 hr).

## 1. Introduction

Depression is a chronic, recurring, and potentially life-threatening illness that affects up to 20% of the population across the globe [1, 2]. This disease is one of the top ten causes of morbidity and mortality worldwide and represents a high cost to country's economy [2]. Available therapy for depression treatment is often associated with several undesirable side effects, and its effectiveness achieves only a certain portion of the population [3]. Therefore, the identification of the alternative therapeutic tools for the treatment of depression is of high importance.

Venlafaxine hydrochloride, (±)-1-[2-(dimethylamino)-1-(4-methoxyphenyl)ethyl] cyclohexanol hydrochloride, is a highly water soluble and structurally novel antidepressant for oral administration. It is a dual serotonin and norepinephrine reuptake inhibitor (SNRI). It inhibits the serotonin transporter at 30-fold lower concentration than norepinephrine transporter (Ki = 82 and 2480 nm), respectively [4]. It displays differential effects on norepinephrine reuptake in healthy versus depressed patients [5]. It is highly soluble in 0.1 N HCl; its solubility decreases with increasing pH over the physiological range. Both venlafaxine and its active metabolite, ODV (O-desmethyl venlafaxine), have weak inhibitory effect on the reuptake of dopamine but, unlike the tricyclics and similar to SSRIs (selective serotonin reuptake inhibitors) they are not active in histaminergic, muscarinic, or alpha(1)-adrenergic receptors [6–9]. The half-life of venlafaxine hydrochloride is 5 ± 2 hr, necessitating the administration, two or three times daily to maintain adequate plasma drug concentration.

Various attempts have been made to develop floating system to control drug release; among them is the so called hydrodynamically balanced system (HBS). Floating drug delivery system (FDDS) or hydrodynamically balanced systems (HBS) have a bulk density lower than the gastric fluid and thus remain buoyant in the stomach without affecting the gastric emptying rate for a prolonged period of time [10]. FDDS is suitable for those drugs which are having an absorption window in the stomach or the upper small intestine [11], for drugs, which act locally in the stomach [12], and for drugs that are poorly soluble or unstable in the intestinal fluid [13]; venlafaxine hydrochloride is one drug from the latter category.

Floating dosage forms remain on the surface of gastric fluid because of its relatively lower density than that of gastric fluid. Floating single unit dosage form, also called hydrodynamically balanced systems (HBS), has been extensively studied [14].

Mucoadhesive delivery systems were also proven to be suitable for reduction of transit time of the dosage form through the gastrointestinal tract. Adhesiveness of the dosage form is based on the bioadhesive power of the polymer. Various synthetic as well as natural polymers have been reported for this approach [15].

Venlafaxine hydrochloride is selected as a drug candidate for this study as its bioavailability is low and half-life ranges in 5 ± 2 hr necessitating frequent administration to maintain the adequate plasma level of drug.

The present research endeavor involves development and characterization of newer floating-mucoadhesive tablets of venlafaxine hydrochloride using HPMC K4MCR and Carbopol 934PNF and investigation of the combined effect of these polymers on the floating behavior and* in vitro* release pattern of the drug. Here the synergism effect of mucoadhesion with floatation may increase the gastric retention of drug, hence increasing its bioavailability.

## 2. Materials and Methods

### 2.1. Materials

Venlafaxine hydrochloride was a kind gift from Ranbaxy Research Lab. Ltd. (Gurgaon, Haryana, India). HPMC K4MCR was obtained as a gift sample from Colorcon Asia Pvt. (Goa, India), Carbopol 934PNF from Arihant Trading Co. (Mumbai, India), and lactose and magnesium stearate were procured from Central Drug House (New Delhi, India). Sodium bicarbonate was obtained from SD Fine-Chem Ltd. (Mumbai, India). All other reagents were of analytical grade, which were used in preparation.

### 2.2. Methodology

Venlafaxine hydrochloride floating tablets were prepared by the wet granulation method using hydroxypropyl methyl cellulose (HPMC K4MCR), Carbopol 934P, lactose, and sodium bicarbonate. The compositions of different formulation codes of floating tablets are shown in Tables 1 [Table tab2] [Table tab3]–4.

#### 2.2.1. Preparation of Granules

Granules were prepared by wet granulation method. First of all, the ingredients were accurately weighed. Then accurately weighed quantities of venlafaxine hydrochloride, HPMC K4MCR, lactose, and sodium bicarbonate were mixed homogeneously using glass mortar and pestle. The wet granulation was done with ethanol (95%). Wet mass was passed through a 40-mesh screen and dried in a hot air oven at 40°C over night. The dried granules were sized through 40/60 mesh and blended with Carbopol 934P and magnesium stearate (approximately 1% w/w). Lactose was used as filler and channeling agent. Sodium bicarbonate was used as a gas generating agent [16].

#### 2.2.2. Evaluation of Granules

Granules of different formulation codes are evaluated for angle of repose, flow rate, bulk density, tapped density, Carr's index, and so forth as per the method described by Aulton [17]. The angle of repose and flow rates were determined by the funnel method. The bulk density and tapped density were obtained by the cylinder method. Consider

(1) $$\begin{array}{c} \left(\;\text{angle of repose}\;\right)\theta =\tan^{-1}\left(\;\frac{h}{r}\;\right), \end{array}$$

where *θ* is the angle of repose, *h* is height of the cone (or pile height), and *r* is radius of cone (or pile) or base radius:

(2) flow rate=weight of granulestime in seconds,bulk densityρb=wVb,bulk densityρt=wVt,

where *w* is weight of the sample in grams, *V*_*b*_ is final bulk volumes of granules in cm^3^, and *V*_*t*_ is final tapped volumes of granules in cm^3^.

And Carr's index of each formulation was calculated according to the equation given below:

(3) $$\begin{array}{c} \text{Carr's index}\left(\;\%\text{ compressibility}\;\right)=\frac{\rho_{t}-\rho_{b}}{\rho_{t}}\times 100. \end{array}$$

Experimental evaluations of granules are shown in Table 5.

#### 2.2.3. Preparation of Floating Tablet

The homogeneously lubricated granules with magnesium stearate (approximately 1% w/w) were then compressed into tablets using nine mm die/punch set on a single punch tablet compression machine (Cadmach Machinery Ltd., Ahmedabad, India). Compression force was adjusted to obtain tablets with hardness in the range of 6.2–6.9 kg/cm^2^ on a Monsanto tablet hardness tester.

#### 2.2.4. Characterization of Floating Tablet

The prepared floating tablets were characterized for drug content, uniformity of weight using 20 tablets, hardness (a Monsanto hardness tester), and friability (Roche type friabilator). The drug content of the tablet was determined using 0.1 N HCl as a solvent. The uniformity of drug content in each formulation was determined by triturating 20 tablets and powder equivalent to average weight was added to 100 mL of 0.1 N hydrochloric acid, followed by stirring for 30 minutes [18]. The solution was filtered through a Whatman filter paper number 41 and diluted suitably and the absorbance of resultant solution was measured using Double Beam UV spectrophotometer (Shimadzu, UV-1700, Japan) at 225.0 nm using 0.1 N hydrochloric acid as blank. The average drug content is calculated.

#### 2.2.5. Floating Characteristics

Floating characteristics of the tablets were studied at the temperature 37 ± 0.5°C, in 250 mL of a 0.1 N HCl solution (pH = 1.2) (Figure 9). The time required for the tablet to rise to the surface of the solution, and to float, was taken as the floating lag time. The duration of time in which dosage form constantly remained on the surface of the medium was considered as total floating time (Table 9).

#### 2.2.6. *In Vitro* Dissolution Studies

The* in vitro* dissolution studies of venlafaxine hydrochloride from floating tablet were carried out using USP Dissolution Testing Apparatus II (Paddle Type). The dissolution test was performed using 900 mL of 0.1 N HCl, at 37 ± 0.5°C at 75 rpm. One mL of the aliquot was withdrawn from the dissolution apparatus at different predetermined time intervals (1, 2, 3, 4, 5, 6, 8, 10, 12, 14, 16, and 18 hours) and the samples were replaced with the fresh dissolution medium. Then the sample was filtered through the Whatman filter paper number 41 and analyzed at 225 nm using UV Spectrophotometer (Shimadzu UV-1700, Japan). Cumulative % drug release was calculated using “PCP Disso v2.08” software.

#### 2.2.7. Mechanism of Drug Release

The drug release mechanism was determined by fitting the data to the various kinetic equations such as zero-order, first-order, Higuchi, and Korsmeyer-Peppas and finding the *R* and *n* values of the release profile corresponding to each model.

#### 2.2.8. Swelling Properties (Hydration Behavior of Matrix Tablets)

The swelling of the polymer can be measured by their ability to absorb water and swell. The swelling property of the formulation was determined by various techniques; here the hydration behavior or swelling properties were determined as per the method described by Deshpande et al. [19].

The swelling properties of HPMC and Carbopol polymer matrices containing the drug were determined by placing the tablet matrices in the USP dissolution test apparatus II. The medium used was 0.1 N HCl, 900 mL rotated at 75 rpm. The medium was maintained at 37 ± 0.5°C throughout the study. The tablets were removed periodically from the dissolution medium. After draining free water these were measured for weight gain, thickness, and diameter. Swelling characteristics were expressed in terms of percentage water uptake (WU%) according to the following equation:

(4) WU%=wt. of swollen tablet−initial wt. of the tabletinitial wt. of the tablet×100.

#### 2.2.9. Detachment Stress

Pieces of sheep fundus tissues were brought from market and stored frozen in saline solution and thawed to room temperature immediately before use. At the time of testing a section of tissue (I) was transferred, keeping the mucosal side out, to the upper glass vial (G) using a rubber band and an aluminum foil cap. The diameter of each exposed mucosal membrane was 1.1 cm. The vials with the fundus tissue were stored at 37°C for 10 min. Next, one vial with a section of tissue (I) was connected to the balance (B), and the other vial was fixed on a height-adjustable pan (P). A bioadhesive tablet (F) was applied to the lower vial with the help of two pieces of adhesive tape. The height of the vial was adjusted so that the tablet could adhere to the mucosal tissues in the vial. The mucoadhesive forces of the floating tablets were determined by the measuring device shown in Figure 5.

A constant weight (10 g) was placed on the upper vial and applied for two minutes, after which it was removed and the upper vial was then connected to the balance. Weights (W) were added at a constant rate to the pan on the other side of the modified balance of the device until the two vials were separated.

The bioadhesive force, expressed as the detachment stress in dyne/cm^2^, was determined from the minimum weight required to detach the two vials using the following equation:

(5) $$\begin{array}{c} \text{detachment stress}\left(\;\text{dyne}/{\text{cm}}^{2}\;\right)=\frac{mg}{A}, \end{array}$$

where *mg* is the weight required for detachment and *A* is the surface area of the tablet [20]. The detachment stresses of the various formulation codes are given in Table 8.

#### 2.2.10. Scanning Electron Microscopy (SEM)

The surface topography of the floating tablet was examined using scanning electron microscopy (Philips FEI XL-30) at different magnification and the acceleration voltage of 14.9 KV. Tablet samples were mounted on a scanning electron microscope holder (aluminum sample mount) using a double side adhesive tape and coated with gold palladium under vacuum and then surface topography was investigated.

#### 2.2.11. Stability Studies

Stability studies were carried out according to ICH and WHO guidelines to assess the drug and formulation stability. The prepared floating tablets of optimized formulation (FC8) containing hydroxypropyl methyl cellulose (HPMC K4MCR) and Carbopol 934PNF (FC8) were selected for stability studies based on* in vitro* drug release, floating lag time, total floating time, and their physical properties. The selected tablets of venlafaxine hydrochloride (FC8) were sealed in aluminum foil packaging coated inside with polyethylene and kept in a humidity chamber at 45°C and 75% RH for three months [21]. At the end and during the study, samples were analyzed for the drug content,* in vitro* dissolution, floating behavior, and other physicochemical parameters (Table 9).

#### 2.2.12. Tablet Preparation for* In Vivo* Studies

The optimized formulation FC8 had shown good* in vitro* buoyancy and sustained-release behavior and hence was finally selected for* in vivo* study (i.e., radiography). The tablets of nine mm diameter and 350 mg in weight were prepared. To make the tablet X-ray opaque, incorporation of BaSO_4_ was necessary. Barium sulphate (BaSO_4_) has high relative density (4.4777 g/cm^3^) and poor floating properties. The amount of the X-ray opaque material in these tablets was sufficient to ensure visibility by X-rays, but at the same time this amount of barium sulphate was low enough to enable tablets to float. For this purpose, the amount of venlafaxine hydrochloride in the formulation, FC8, was replaced with 40 mg of barium sulphate, and all other ingredients were kept constant. These tablets were analyzed for hardness and floating properties. The analysis confirmed that these tablets were similar to the tablets for* in vitro* testing.

#### 2.2.13. *In Vivo* Radiographic Studies

Nowadays, radiographic study is a very popular evaluation parameter for floating dosage form. It helps to locate dosage form in the GIT by which one can predict and correlate the gastric retention time, floating behavior, and passage of the dosage form in the GIT. Here the inclusion of radio-opaque material into a solid dosage form enables it to be visualized by X-rays. It is also important that dosage forms are nondisintegrating units, and animal subjects are young and healthy [22].

The animal protocol to carry out* in vivo* radiographic studies was reviewed and approved by the Institutional Animals Ethical Committee of Bundelkhand University, India (registration number 716/02/a/CPCSEA). The* in vivo* radiographic studies were conducted in young and healthy male albino rabbits weighing 2.0 to 2.2 kg. The animals were kept under standard laboratory conditions (temperature: 25 ± 2°C). Rabbits were kept for one week in the animal house to acclimatize them and fed a fixed standard diet. The 4 healthy male albino rabbits were used to monitor the* in vivo* transit behavior of the floating tablet. None of them had symptoms or history of gastrointestinal (GI) disease. In order to standardize the conditions of GI motility, the animals were fasted for 12 hours prior to the commencement of each experiment. In each experiment, the first radiograph of the animal subjects was made to ensure the absence of radio-opaque material in the GIT. One of the tablets prepared for radiography was orally administered to rabbits with the sufficient amount of water. During the study the rabbits were not allowed to eat, but water was available* ad libitum*.

For radiographic imaging, all four legs of the rabbit were tied over a piece of plywood (20 × 20 inch), and location of the formulation in the stomach was monitored by keeping the subjects in front of X-ray machine (Allengers, Bharat Electricals, India, Model number E 080743). The distance between the source of X-rays and the object was the same for all the imaging. This allowed us to see the tablet in the body of the stomach, antrum, and/or pyloric part of the stomach so that observations of the tablet movements could be made. Gastric radiography was done at 1 hr, 3 hr, and 6 hr. In between the radiographic imaging, the animals were freed and allowed to move and carry out normal activities but were not allowed to take any food. The mean gastric residence time of the drug was calculated.

## 3. Results and Discussion

The granules prepared for the compression of floating tablets were evaluated for their flow properties (Table 5). Granules of matrix tablets of different formulation codes showed the angle of repose from 22.29° ± 0.85 to 37.39° ± 1.73 and flow rates from 0.91 ± 0.13 to 1.22 ± 0.10 gm/sec and Carr's index was found in the range of 18.13 to 26.53. These results indicate that as the concentration of the HPMC K4MCR and Carbopol 934PNF increased in the formulations the angle of repose and Carr's index were found to be increased while their flow rate was found to be decreased. Thus the angle of repose and Carr's index value of different batches of granules indicate satisfactory flow behavior. Other granules parameters were also determined and found to be within acceptable limits.

The floating tablets of venlafaxine hydrochloride were prepared by wet granulation method using HPMC K4MCR, Carbopol 934PNF, sodium bicarbonate, and lactose. The magnesium stearate was used as the lubricant.

The optimization of the formulation was done based on adjusting the drug-polymer ratio, floating lag time, duration of floating, gas generating agents,* in vitro* drug release rate.

**Figure 1:**
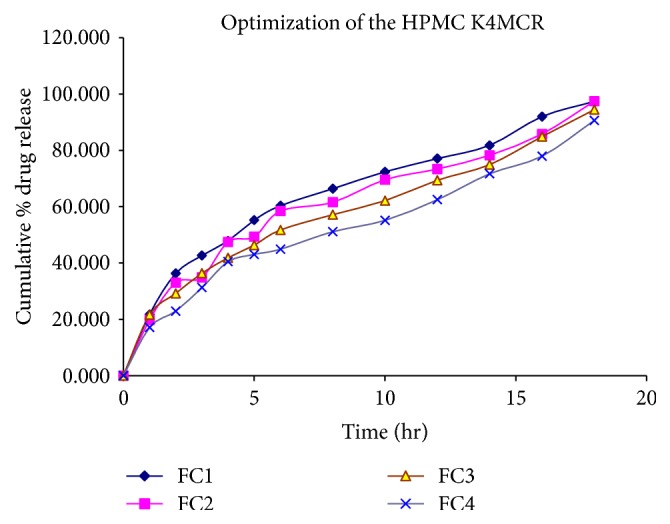
Release profile of venlafaxine hydrochloride in 0.1 N HCl (pH 1.2) for formulation codes FC1, FC2, FC3, and FC4.

**Figure 2:**
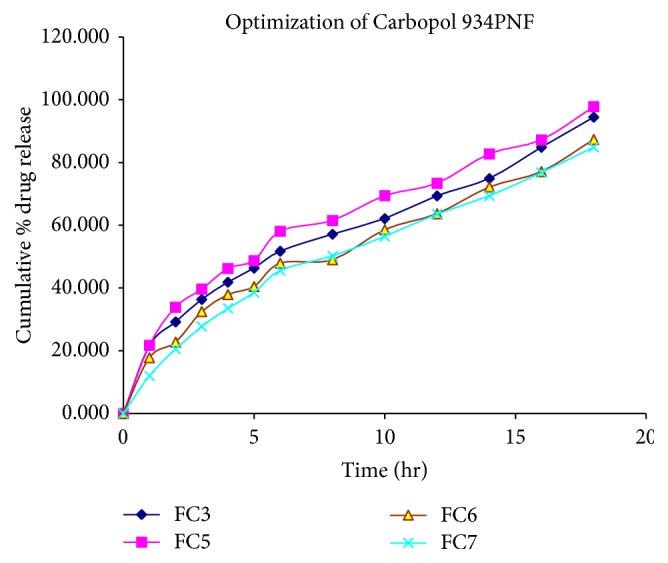
Release profile of venlafaxine hydrochloride in 0.1 N HCl (pH 1.2) for formulation codes FC3, FC5, FC6, and FC7.

Results indicated that the low-density polymers like HPMC K4MCR and Carbopol 934PNF affects the floating behavior of the venlafaxine hydrochloride floating tablet. HPMC K4MCR and Carbopol 934PNF are selected because of their high viscosities, which are desired for the sustained release. For optimizing the concentration ratio of the drug, polymers and several formulations of the different batches were formulated randomly in which only drug to the polymer ratio varied and remaining ingredients of formulation remained constant. Then floating behavior and release rate study of these formulations were determined in the 0.1 N HCl (pH 1.2). Then a certain ratio of drug to polymer gives satisfactory floating behavior and* in vitro* drug release in 0.1 N HCl. At this stage of optimization, few batches of formulation codes FC1, FC2, FC3, and FC4 were prepared for the optimizing concentration of the polymer for obtaining the best floatation behavior and* in vitro* drug release. Floating lag time and duration of floating was found to be 112, 99, 88, and 74 seconds and approximately 17, 18, 23, and 27 hr, respectively. The cumulative % drug release was found to be 97.34, 97.43, 94.43, and 90.65 in 18 hr for the formulation codes FC1, FC2, FC3, and FC4 (Figure 1). But the release pattern of the FC3 showed better* in vitro* release of drug in the sustained manner in predetermined time intervals than formulations FC1, FC2, and FC4. Formulation FC3 also showed better floatation behavior (i.e., floating lag time 88 seconds and duration of floating approximately 23 hr). Therefore, formulation FC3 has been selected in order to obtain the optimum HPMC loading level, because it has better* in vitro* release pattern and floatation behavior. Results also indicate that as the concentration of the HPMC increased, the cumulative % drug release behavior and floating lag time were also decreased to a certain value. After optimizing the concentration of HPMC K4MCR, next optimization was done for Carbopol concentration. As the concentration of the Carbopol in the FC5 formulation decreased, then the* in vitro* drug release pattern of the formulation (FC5) was increased compared to that of the FC3 formulation for 18 hr dissolution study. It was found that as the concentration of the Carbopol increased in the formulation (i.e., FC6 (20 mg) and FC7 (25 mg)), then* in vitro* drug release was also decreased compared to that of FC3 (15 mg). Because the release and floatation behavior exhibited by the FC3 were much better than FC5, FC6, and FC7 (Figure 2), the formulation FC3 remained selected for the further study.

**Figure 3:**
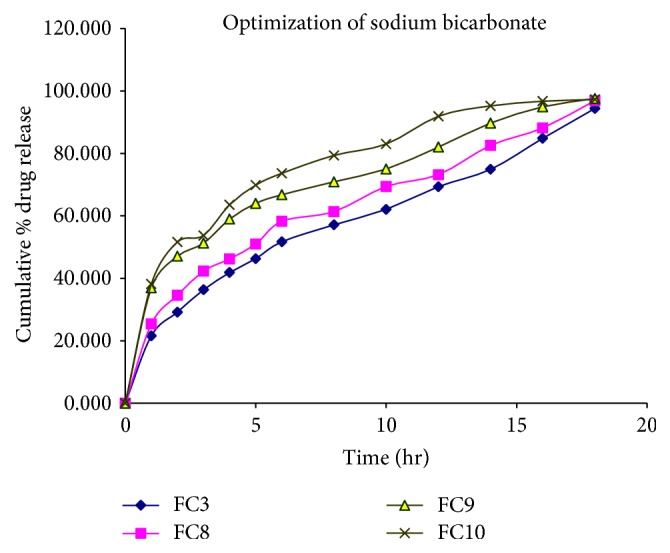
Release profile of venlafaxine hydrochloride in 0.1 N HCl (pH 1.2) for formulation codes FC3, FC8, FC9, and FC10.

For the floating drug delivery system, the ideal matrix should be highly permeable for the dissolution media in order to initiate rapid generation of the carbon dioxide gas (CO_2_) and also should be permeable for CO_2_ to promote floating. The buoyancy lag time of the tablets depends on the concentration of the sodium bicarbonate (NaHCO_3_) involved in the CO_2_ formation. In the trial study,* in vitro* release of drug from the different formulation batches was studied to determine the optimum concentration of the gas generating agent. It was observed from the formulations FC8, FC9, and FC10 that as the concentration of the sodium bicarbonate increased in these formulations, the floating lag time was found to be decreased, but their corresponding release of drug was increased (Figure 3). The trial study also showed that, at higher concentration of the sodium bicarbonate, FC10 (30 mg) causes bursting and rapid disintegration of tablet. Hence it was desirable to use optimum concentration of sodium bicarbonate to get the least floating lag time with minimum bursting effect and desired floating time without rapid disintegration. So FC8 with sodium bicarbonate (20 mg) was optimized and selected to achieve optimum* in vitro* buoyancy with less bursting effect. The formulation FC8 was preferred over the FC3, because the* in vitro* release of drug was increased in the more controlled manner than that of FC3.

**Figure 4:**
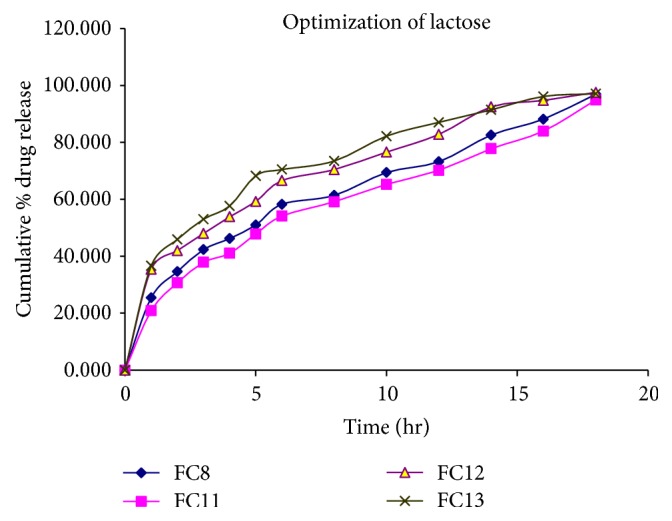
Release profile of venlafaxine hydrochloride in 0.1 N HCl (pH 1.2) for formulation codes FC8, FC11, FC12, and FC13.

**Figure 5:**
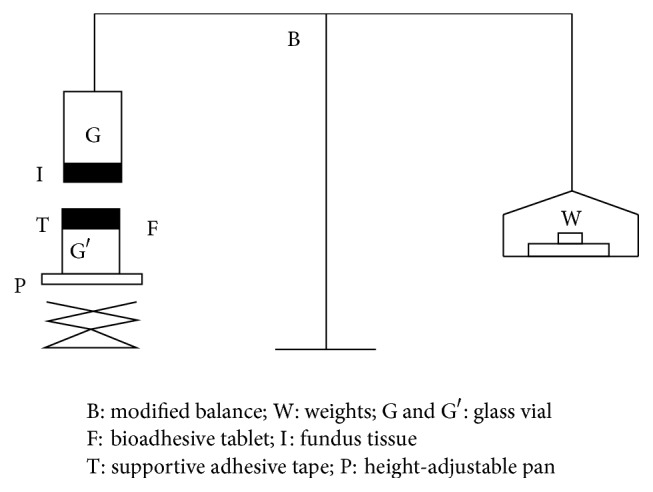
Mucoadhesion measuring device for obtaining detachment stress.

Here, the lactose acts as channeling agent which is soluble in the dissolution medium; therefore, the matrix integrity gets broken and showed faster* in vitro* drug dissolution. Results indicate from the formulations (i.e., FC11, FC12, and FC13) that release rate of drug is directly proportional to the concentration of the lactose (Figure 4). As the concentration of the lactose increased,* in vitro* drug releases were also found to be increased but if the concentration of the lactose increased more than 50 mg then the tablet will start disintegration by the erosion mechanism. Therefore, optimum concentration of the lactose is required for getting the maximum* in vitro* drug release without causing erosion. From the above results, FC8 was found to be the best formulation which showed better* in vitro* drug release and floatation behavior.

The diameter of the formulations FC1 to FC13 was found in the range of 8.97 ± 0.01 to 8.99 ± 0.03 and thickness was in the range of 3.96 ± 0.01 to 3.99 ± 0.02. The variation in the weight was found in the range of ±5% complying with pharmacopoeial specifications. The hardness of different formulation was found to be between 6.27 ± 0.41 and 6.93 ± 0.52 kg/cm^2^ indicating good mechanical strength. The friability was below 1% for all formulations which is an indication of good mechanical resistance of tablet. The drug content varied between 98.00% and 99.21% in different formulation with low standard deviation (Table 6).

The swelling of the polymers used (i.e., HPMC K4MCR and Carbopol 934PNF) was determined by swelling index of the tablet. Hydrophilic matrices (i.e., HPMC K4MCR and Carbopol 934PNF) in contact with water swell and increase their volume due to water diffusion through the matrix. The polymer chains continue the hydration process, and the matrix gained more water. Drug diffusion significantly depends on the water content of the tablet. This may be because the mobility of the polymer chains is very dependent on the water content of the system. In case of high-water content, polymer chain relaxation takes place with volume expansion resulting in marked swelling of the system. Also, higher water content could lead to greater penetration of the gastric fluid into the tablet leading to quicker carbon dioxide gas generation, thereby reducing the floating lag time. Consequently, quicker and greater swelling of the tablet would lead to an increase in the diffusion pathway and, thus, a reduction in diffusion rate. So the drug release was found to be high initially and then gradually decreased.

The % swelling index of all formulations from FC1 to FC10 at 24 hr was found to be between 136.03 and 170.99, respectively. The percentage water uptake was found to be improved as the concentration of the HPMC K4MCR and Carbopol 934PNF increased in the formulation. The result indicates, from Table 7, that swelling index was increased much more in case of Carbopol compared to HPMC because Carbopol is bearing very good water sorption property. It was also observed that as the concentration of the Carbopol in the formulation decreased, the % water uptake was also found to be decreased (i.e., FC5).

Table 8 clearly indicates that the value of bioadhesive force was found to be increased significantly as the concentration of the mucoadhesive polymer HPMC and Carbopol increased. All floating formulations showed mucoadhesive forces in the range of 110.42 to 167.80 dynes/cm^2^. Results indicate that as the concentration of HPMC K4MCR increased in the formulations FC1 to FC4, then the value of detachment forces also increased from 123.45 to 156.72 dynes/cm^2^, respectively. It was also observed that as the concentration of Carbopol 934PNF increased, the values of detachment forces were increased from the 159.65 to 167.00 dynes/cm^2^ for formulations FC6 to FC7, respectively, but as the concentration of Carbopol decreased, value of forces was decreased (i.e., FC5). Hence, it was also concluded from the results that the values of detachment forces were increased much more in case of Carbopol 934PNF compared to HPMC K4MCR, because the Carbopol was highly bioadhesive as compared to HPMC.

Bioadhesion is a surface phenomenon in which a material of synthetic or natural origin adheres or sticks to a biological surface, usually mucus membrane. Many hydrophilic polymers adhere to mucosal surfaces as they attract water from the mucus gel layer adhering to the epithelial surface. This is the simplest mechanism of adhesion, and it has been defined as “adhesion by hydration.” The hydrogen bonding present between the adherent polymer and mucus is involved in mucoadhesion at the molecular level. As the concentration of bioadhesive polymers increased, then the hydrogen bonding between the mucus membrane and adherent polymer at the molecular level increased; therefore, detachment forces were also found to be increased.

**Figure 6:**
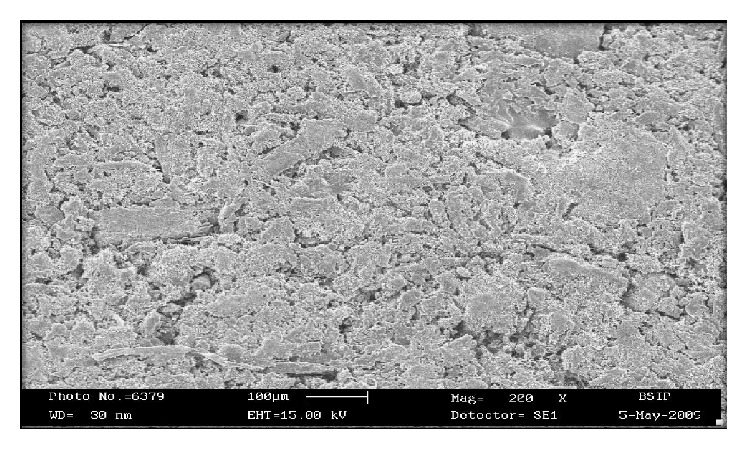
Scanning electron microscopy of optimized formulation FC8 of tablet surface before dissolution.

**Figure 7:**
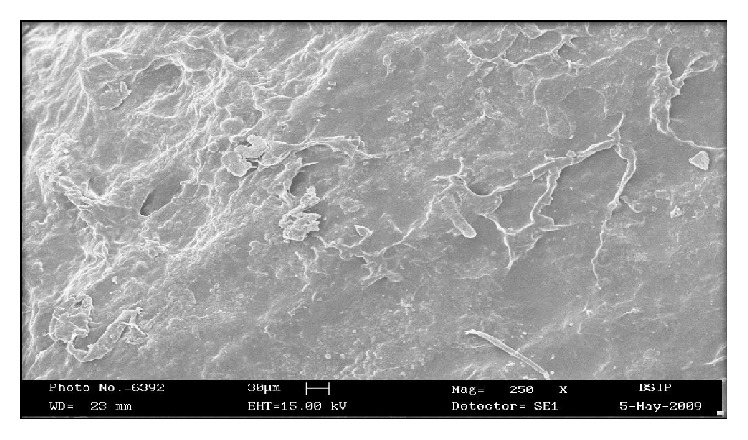
Scanning electron microscopy of optimized formulation FC8 of tablet surface after dissolution.

Surface morphology of the optimized formulation FC8 was examined by scanning electron microscopy (SEM). It was observed from the SEM photographs that the drug is released from the matrix tablet by the diffusion process. The surface morphology of the matrix tablet after dissolution showed that the solvent front enters the matrix and moves slowly toward the center of the tablet. The drug diffuses out of the matrix after it comes in contact with dissolution medium. The images of the tablet showed a network in the swollen polymer through which the drug diffused to the surrounding medium. Thus it was concluded that the drug was released from a matrix by diffusion mechanism (Figures 6 and 7).

**Figure 8:**
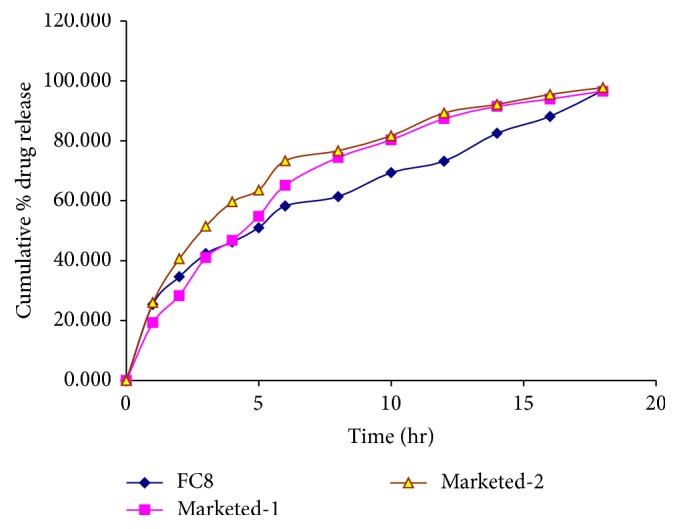
Comparison of % drug release of optimized formulation (FC8) versus marketed formulations for 18 hr.

**Figure 9:**
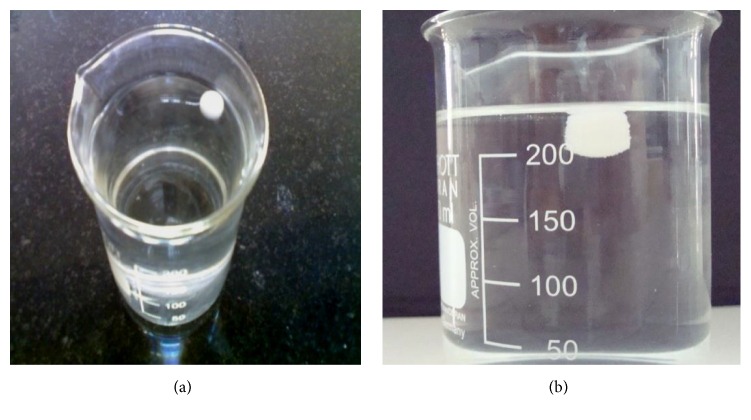
*In vitro* floating studies of optimized formulation (FC8) (a) after 80 sec and (b) after 24 hr.

A comparative study was performed on the available marketed formulation of extended release tablet and capsule containing venlafaxine hydrochloride equivalent to venlafaxine 37.5 mg. The results obtained from the* in vitro* drug release study showed that optimized formulations FC8 showed more sustained-release action than that of tablet (marketed-1) and capsule (marketed-2) with respect to time of 18 hr (Figure 8).

The drug release was found to follow first-order kinetics with *r* = 0.9935 and 0.9904 for marketed-1 (tablet) and marketed-2 (capsule), respectively, while optimized formulation FC8 followed the Peppas model with value of *r* = 0.9962, *n* = 0.4473, and *k* = 25.2788. So the best-fit model was found to be the Peppas model for optimized formulation FC8. The marketed formulations of tablet and capsule showed more than 90% of drug release in 14 hr while optimized formulation FC8 showed only 82.58% of drug release in 14 hr.

The data obtained from* in vitro* dissolution studies were fitted to zero-order, first-order, Higuchi, and Korsmeyer-Peppas equations. All release kinetic models were applied on the formulation codes FC1, FC2, FC3, FC4, FC5, FC6, FC7, FC8, FC9, FC10, FC11, FC12, and FC13 due to their satisfactory release behavior. The best-fit model was found to be the matrix for formulation codes FC1, FC2, and FC5 and Peppas for formulation codes FC3, FC4, FC6, FC7, FC8, FC9, FC10, FC11, FC12, and FC13. The selection criteria for the best model were based on goodness of fit of the data and residual sum of squares. The details of the best-fit model of different formulation codes are given in Table 10.

For confirming the diffusion mechanism of optimized formulation FC8, the data was fitted to the Korsmeyer equation, which showed the exponent value *n* = 0.4473. Because the optimized formulation FC8 had the exponent value (*n*) less than 0.5, it was suggested that the release mechanism of venlafaxine hydrochloride indicates swelling and diffusion mechanism of floating tablets, which followed the Fickian diffusion (Case, I transport). This kinetic data analysis was done by using “PCP Disso V2.08” Software (Poona College of Pharmacy, Pune, India).

The optimized formulation FC8 was suggested for stability study based on* in vitro* floating lag time, total floating time,* in vitro* drug dissolution studies, and its physical properties. The floating tablets were investigated at 400°C/75% RH in aluminum foil packaging for three months. The increased lag time indicates the possibility of reaction of sodium bicarbonate with moisture during the study period. However, there was the very little effect on the duration of the floating and matrix integrity of the tablets. Some drug degradation was found, but it was not statistically significant. Decreased drug release was found from the formulation but drug released compiled the official standard of release, since more than 80% of the drug was released (Table 9). Thus, it was found that the floating tablets of venlafaxine hydrochloride were almost stable under these storage conditions for at least three months.

The* in vitro* studies, with BaSO_4_-containing floating tablets, showed a floating lag time of 112 ± 2.00 seconds, hardness of 6.60 ± 0.03 kg/cm^2^, and thickness of 3.99 ± 0.01 mm. The intragastric behavior of the BaSO_4_-loaded tablet in the albino rabbit was observed by using a radiographic imaging technique. Radiographic images were taken after 1, 3, and 6 hr showing that the tablet had altered its position in the stomach. This provided evidence that the tablets were floating on the gastric fluid. After 4 hr of the tablet administration, the swelling of the tablet is visualized very well together with the white dry core and translucent swelling layer around it due to the swellable polymers, HPMC K4MCR and Carbopol 934PNF.

**Figure 10:**
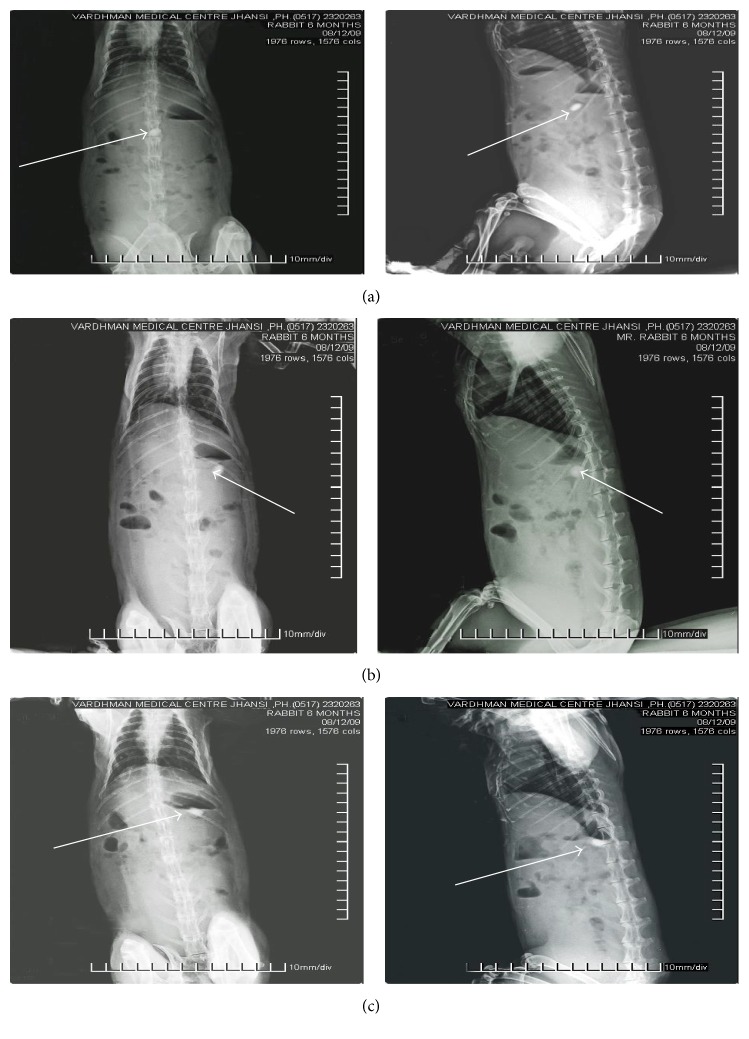
Radiographic images showing the presence of BaSO_4_ loaded floating tablet in the stomach at different time periods (the tablet is indicated with an arrow). Images were taken after (a) 1 hr, (b) 3 hr, and (c) 6 hr, after tablet administration.

Hence, examination of the sequential radiographic images of the GIT during the study clearly indicated that the tablet remained buoyant and altered its position in the gastric contents for more than 6 hr. So prolonged gastric retention time (GRT) of more than 6 hr is achieved (Figures 10(a)–10(c)).

The objective of the* in vivo* studies was to provide the proof of concept that the floating capability of the floating tablet was useful for the increasing the gastric residence time of the dosage form. For facilitating the trials on the rabbits, the dosage form did not contain any drug, and the scope was limited to the observation of the behavior of the system in the stomach.

## 4. Conclusion

The present study was carried out to develop the floating-bioadhesive drug delivery of venlafaxine hydrochloride using HPMC K4MCR and Carbopol 934PNF polymers as the carrier. Optimized formulation FC8 gave satisfactory results for various physiochemical evaluations for tablets like hardness, weight variation, floating lag time, and uniformity of content.* In vitro* dissolution studies of the optimized formulation FC8 showed the sustained release for 18 hr, followed by the Fickian diffusion, and an* in vivo* study indicated increased gastric residence time by the floating principle and was considered desirable for improving the bioavailability of the drug. All these results also indicated that a low amount of floating agent and high amount of hydrophilic polymer favored the sustained release of venlafaxine hydrochloride from the floating gastroretentive tablet formulations. Developed sustained-release oral formulation for prolonged release would be a significant advantage for the patient and may result in fewer side effects due to reduction of the blood concentration fluctuations, especially in long-term therapy. Thus, results of the current study clearly indicate a promising potential of the venlafaxine hydrochloride floating system as an alternative to the conventional dosage form. However, further clinical studies are needed to assess the utility of this system for patients suffering from depression.

## Figures and Tables

**Table 1:** Formulation for optimization of drug: HPMC K4MCR ratio.

Ingredients (in mg)	Formulation code (FC)			
	FC1	FC2	FC3	FC4
Venlafaxine hydrochloride	42.43	42.43	42.43	42.43
HPMC K4MCR	**130**	**135**	**140**	**145**
Carbopol 934PNF	15	15	15	15
Sodium bicarbonate	15	15	15	15
Lactose	30	30	30	30
Magnesium stearate	2.57	2.57	2.57	2.57
*Tablet Wt. (mg)*	*235*	*240*	*245*	*250*

**Table 2 tab2:** Formulation for optimization of drug: Carbopol 934PNF ratio.

Ingredients (in mg)	Formulation code (FC)			
	FC3	FC5	FC6	FC7
Venlafaxine hydrochloride	42.43	42.43	42.43	42.43
HPMC K4MCR	140	140	140	140
Carbopol 934PNF	**15**	**10**	**20**	**25**
Sodium bicarbonate	15	15	15	15
Lactose	30	30	30	30
Magnesium stearate	2.57	2.57	2.57	2.57
*Tablet Wt. (mg)*	*245*	*240*	*250*	*255*

**Table 3 tab3:** Formulation for optimization of drug: sodium bicarbonate ratio.

Ingredients (in mg)	Formulation code (FC)			
	FC3	FC8	FC9	FC10
Venlafaxine hydrochloride	42.43	42.43	42.43	42.43
HPMC K4MCR	140	140	140	140
Carbopol 934PNF	15	15	15	15
Sodium bicarbonate	**15**	**20**	**25**	**30**
Lactose	30	30	30	30
Magnesium stearate	2.57	2.57	2.57	2.57
*Tablet Wt. (mg)*	*245*	*250*	*255*	*260*

**Table 4:** Formulation for optimization of drug: lactose ratio.

Ingredients (in mg)	Formulation code (FC)			
	FC8	FC11	FC12	FC13
Venlafaxine hydrochloride	42.43	42.43	42.43	42.43
HPMC K4MCR	140	140	140	140
Carbopol 934PNF	15	15	15	15
Sodium bicarbonate	20	20	20	20
Lactose	**30**	**20**	**40**	**50**
Magnesium stearate	2.57	2.57	2.57	2.57
*Tablet Wt. (mg)*	*250*	*240*	*260*	*270*

**Table 5:** Comparative study of various granules characteristics.

Formulation code	Angle of repose (*θ*°)	Flow rate (gm/second)	Bulk density (gm/cm^3^)	Tapped density	Carr's index
FC1	24.70 ± 1.82	1.18 ± 0.02	0.672 ± 1.26	0.826 ± 1.06	18.64
FC2	25.98 ± 1.74	1.13 ± 0.17	0.651 ± 0.28	0.817 ± 1.02	20.32
FC3	26.89 ± 1.02	0.98 ± 0.11	0.681 ± 0.40	0.887 ± 0.80	23.22
FC4	28.12 ± 1.16	0.91 ± 0.13	0.648 ± 0.16	0.862 ± 0.50	24.82
FC5	31.09 ± 0.82	1.10 ± 0.12	0.624 ± 1.08	0.801 ± 0.18	22.10
FC6	31.47 ± 1.11	0.99 ± 0.09	0.667 ± 0.30	0.907 ± 1.20	26.46
FC7	37.39 ± 1.73	0.95 ± 0.05	0.662 ± 0.24	0.901 ± 0.30	26.53
FC8	32.15 ± 1.04	0.98 ± 0.03	0.652 ± 1.01	0.807 ± 1.08	19.21
FC9	27.08 ± 1.32	1.07 ± 0.07	0.664 ± 0.36	0.823 ± 0.45	19.32
FC10	30.07 ± 1.51	1.05 ± 0.19	0.694 ± 0.50	0.852 ± 0.16	18.54
FC11	31.09 ± 0.86	1.21 ± 0.16	0.701 ± 0.30	0.905 ± 0.10	22.54
FC12	26.06 ± 0.87	1.22 ± 0.10	0.721 ± 0.18	0.910 ± 1.23	20.77
FC13	22.29 ± 0.85	1.20 ± 0.08	0.736 ± 0.10	0.899 ± 0.35	18.13

Mean ± SD; *n* = 3; FC: formulation code.

**Table 6:** Physiochemical characterization of venlafaxine hydrochloride tablets.

Code	% weight variation	Hardness (kg/cm^2^)	% friability	% drug content uniformity	Floating lag time (sec)	Total floating time (hr)
FC1	±3.0%	6.40 ± 0.16	0.524	99.21 ± 1.46	112 ± 2.87	17.33 ± 2.05
FC2	±4.5%	6.37 ± 0.45	0.564	98.47 ± 0.34	99 ± 2.49	18.50 ± 1.87
FC3	±3.5%	6.53 ± 0.57	0.572	98.86 ± 1.24	88 ± 2.87	23.33 ± 0.62
FC4	±4.0%	6.63 ± 0.58	0.482	97.20 ± 0.18	74 ± 3.27	27.08 ± 0.95
FC5	±2.5%	6.93 ± 0.52	0.458	98.53 ± 0.52	94 ± 3.27	12.75 ± 2.08
FC6	±3.0%	6.70 ± 0.70	0.545	99.08 ± 2.21	82 ± 2.94	26.50 ± 1.02
FC7	±4.0%	6.43 ± 0.33	0.584	97.40 ± 1.02	76 ± 2.45	29.25 ± 0.74
FC8	±4.0%	6.67 ± 0.98	0.580	98.91 ± 0.18	72 ± 2.49	24.50 ± 0.74
FC9	±2.5%	6.70 ± 0.49	0.495	98.00 ± 0.24	64 ± 2.94	26.50 ± 1.54
FC10	±3.0%	6.47 ± 0.90	0.457	97.56 ± 1.32	54 ± 2.45	26.25 ± 1.87
FC11	±3.0%	6.80 ± 0.43	0.530	98.43 ± 0.64	67 ± 3.74	28.25 ± 0.69
FC12	±4.5%	6.27 ± 0.41	0.572	99.02 ± 1.45	81 ± 2.87	20.52 ± 0.92
FC13	±4.5%	6.67 ± 0.66	0.592	98.74 ± 0.82	93 ± 3.27	18.00 ± 0.82

Mean ± SD; *n* = 3; FC: formulation code.

**Table 7:** Swelling behavior of matrix tablets.

Formulation code	Initial weight (mg)	Initial thickness (mm)	Initial diameter (mm)	Final weight (mg)	Final thickness (mm)	Final diameter (mm)	Swelling index
FC1	234.11 ± 3.05	3.97 ± 0.01	8.98 ± 0.01	552.59 ± 3.11	4.42 ± 1.08	18.16 ± 0.38	136.03
FC2	240.20 ± 4.35	3.98 ± 0.02	8.97 ± 0.02	578.52 ± 1.32	4.50 ± 0.74	18.52 ± 1.20	140.84
FC3	244.18 ± 1.69	3.97 ± 0.01	8.99 ± 0.01	607.10 ± 5.06	4.56 ± 0.89	18.73 ± 1.48	148.67
FC4	250.10 ± 1.71	3.99 ± 0.02	8.97 ± 0.01	637.22 ± 3.40	4.61 ± 1.21	18.85 ± 0.92	154.78
FC5	239.58 ± 1.01	3.98 ± 0.01	8.98 ± 0.02	578.48 ± 2.25	4.53 ± 1.42	18.62 ± 1.34	141.45
FC6	250.09 ± 1.89	3.96 ± 0.01	8.99 ± 0.02	648.86 ± 4.13	4.63 ± 1.27	18.89 ± 0.88	159.45
FC7	255.58 ± 3.29	3.97 ± 0.02	8.97 ± 0.01	681.68 ± 2.67	4.66 ± 0.54	18.93 ± 1.53	166.72
FC8	250.17 ± 1.23	3.99 ± 0.02	8.99 ± 0.01	654.25 ± 3.28	4.62 ± 0.28	18.99 ± 0.67	161.52
FC9	254.21 ± 3.53	3.98 ± 0.01	8.98 ± 0.01	678.17 ± 1.44	4.64 ± 0.78	19.14 ± 0.25	166.78
FC10	259.67 ± 4.42	3.99 ± 0.02	8.97 ± 0.02	703.67 ± 2.87	4.68 ± 0.38	19.27 ± 1.44	170.99

Mean ± SD; *n* = 3; FC: formulation code.

**Table 8:** Detachment forces of different formulations.

S. number	Formulation code (FC)	Detachment force (dynes/cm^2^)
1	FC1	123.45
2	FC2	131.74
3	FC3	147.53
4	FC4	156.72
5	FC5	110.42
6	FC6	159.65
7	FC7	167.80
8	FC8	142.18
9	FC9	140.21
10	FC10	136.24
11	FC11	143.78
12	FC12	145.26
13	FC13	140.65

**Table 9:** Stability studies of venlafaxine hydrochloride floating tablets of optimized formulation (FC8).

Characteristic	Initial	1st month	2nd month	3rd month
Hardness (kg/cm^2^)	6.67 ± 0.98	6.02 ± 0.56	6.42 ± 0.20	6.30 ± 0.88
Drug content (%)	98.91 ± 0.18	98.34 ± 0.02	98.12 ± 1.42	98.00 ± 0.27
Floating lag time (seconds)	72 ± 2.65	76 ± 1.26	84 ± 2.45	90 ± 3.26
Total floating time (hours)	24.50 ± 0.74	24.16 ± 1.12	24.18 ± 2.26	24.08 ± 0.34
Swelling index (%)	161.52	160.82	160.64	160.18
Buoyancy on disturbing	Float	Float	Float	Float
Matrix integrity	Very good	Very good	Very good	Very good
*% in vitro* release 18 hours	96.92 ± 0.83	94.43 ± 1.38	93.98 ± 0.42	93.00 ± 1.56

Mean ± SD; *n* = 3; FC: formulation code.

**Table 10:** Drug release kinetics for floating-bioadhesive tablets.

S. number	Formulation code (FC)	Best-fit model	*R* value	Parameters for Korsmeyer-Peppas equation
1	FC1	Matrix	0.9948	*n* = 0.4817
				*k* = 24.2487

2	FC2	Matrix	0.9942	*n* = 0.5125
				*k* = 21.3988

3	FC3	Peppas	0.9966	*n* = 0.4919
				*k* = 21.0756

4	FC4	Peppas	0.9914	*n* = 0.5500
				*k* = 16.9021

5	FC5	Matrix	0.9968	*n* = 0.4877
				*k* = 22.9187

6	FC6	Peppas	0.9941	*n* = 0.5430
				*k* = 17.1357

7	FC7	Peppas	0.9966	*n* = 0.6472
				*k* = 13.0990

8	FC8	Peppas	0.9962	*n* = 0.4473
				*k* = 25.2788

9	FC9	Peppas	0.9942	*n* = 0.3266
				*k* = 37.4233

10	FC10	Peppas	0.9910	*n* = 0.3279
				*k* = 39.6415

11	FC11	Peppas	0.9972	*n* = 0.4974
				*k* = 21.3024

12	FC12	Peppas	0.9933	*n* = 0.3702
				*k* = 33.2823

13	FC13	Peppas	0.9934	*n* = 0.3466
				*k* = 36.6938

Note: each sample was analyzed in triplicate (*n* = 3).

*k*: kinetic constant.

*n*: exponent value.
